# Pre-amplification methods for tracking low-grade *Plasmodium falciparum* populations during scaled-up interventions in Southern Zambia

**DOI:** 10.1186/1475-2875-13-89

**Published:** 2014-03-12

**Authors:** Sungano Mharakurwa, Rachel Daniels, Alan Scott, Dyann F Wirth, Philip Thuma, Sarah K Volkman

**Affiliations:** 1 Malaria Institute at Macha, Choma, Zambia; 2 Johns Hopkins Bloomberg School of Public Health, Baltimore, MD, USA; 3 Harvard School of Public Health, Boston, MA, USA; 4 The Broad Institute, Cambridge, MA, USA; 5 Simmons College, Boston, MA, USA

**Keywords:** *Plasmodium falciparum*, Genotyping, Pre-amplification, Asymptomatic, Rapid diagnostic test, Diagnostics, Southern Africa, Zambia

## Abstract

**Background:**

Malaria is receding in many endemic countries with intervention scale -up against the disease. However, this resilient scourge may persist in low-grade submicroscopic infections among semi-immune members of the population, and be poised for possible resurgence, creating challenges for detection and assessment of intervention impact. Parasite genotyping methods, such as the molecular barcode, can identify specific malaria parasite types being transmitted and allow tracking and evaluation of parasite population structure changes as interventions are applied. This current study demonstrates application of pre-amplification methods for successful detection and genotyping of residual *Plasmodium falciparum* infections during a dramatic malarial decline.

**Methods:**

The study was a prospective cross-sectional design and based on a 2,000 sq km vicinity of Macha Mission Hospital in southern Zambia. Willing and predominantly asymptomatic residents of all ages were screened for malaria by microscopy during the 2005 and 2008 transmission seasons, with simultaneous collection of dried blood spots (DBS) on filter paper, and extraction of *Plasmodium falciparum* DNA was performed. *Plasmodium falciparum* infections were genotyped using a 24 SNP-based molecular barcode assay using real-time PCR. Submicroscopic parasitaemia samples were subjected to pre-amplification using TaqMan PreAmp Master Mix following the manufacturer’s instructions before SNP barcode analysis.

**Results:**

There was a dramatic decline of malaria between 2005 and 2008, and the geometric mean parasite density (95% CI) fell from 704/μL (390–1,271) in 2005 to 39/μL (23–68) in 2008, culminating in a large proportion of submicroscopic infections of which 90% failed to yield ample DNA for standard molecular characterization among 2008 samples. Pre-amplification enabled successful detection and genotyping of 74% of these low-grade reservoir infections, overall, compared to 54% that were detectable before pre-amplification (*p* <0.0005, n = 84). Furthermore, nine samples negative for parasites by microscopy and standard quantitative PCR amplification were positive after pre-amplification.

**Conclusions:**

Pre-amplification allows analysis of an otherwise undetectable parasite population and may be instrumental for parasites identification, tracking and assessing the impact of interventions on parasite populations during malaria control and elimination programmes when parasitaemia is expected to decline to submicroscopic levels.

## Background

Through support from the WHO Global Fund Rollback Malaria (RBM) programme, the President’s Malaria Initiative (PMI), Malaria Control and Evaluation Partnership (MACEPA) and other public-private partnerships, endemic countries are scaling up effective interventions against malaria [1-4]. Declines in the malaria burden have since been reported throughout sub-Saharan Africa [4]. Many countries, especially those situated towards the natural fringes of malaria transmission, such as the southern African region, are aiming for possible elimination of the disease.

However, the malaria decline has not been universal, both between and within countries [5], posing risks of resurgence from this formidably resilient disease. Tracking changes in parasite population structure as it invariably adapts to interventions is not only instrumental in validating the impact of interventions on silent and non-silent reservoirs, but also in devising strategies for better targeting the scourge until its eventual elimination. As the parasite is brought under control, one of its adaptive strategies is to persist as silent low-grade carriage. Harboured in semi-immune members of the community, the predominantly submicroscopic infections evade not only detection but also treatment, while presumably capitalizing on the opportunity for genomic adaptation to overcome interventions. This asymptomatic reservoir is believed to contribute to new infections and will need to be identified and addressed for malaria elimination to occur. The present study applies a pre-amplification technique and demonstrates its utility for enhanced molecular detection and tracking of recalcitrant *Plasmodium falciparum* populations during scaled up interventions.

## Methods

### Study area and population

The study was based in a 2,000 sq km vicinity of the Malaria Institute at Macha (MIAM). This area is intrinsically hyperendemic for malaria and is transitioning to hypoendemicity under scaled up interventions against the disease. The resident population comprises essentially stable subsistence farming communities of the Batonga tribe. Ethical approval was obtained for the use of these samples for parasite genotypic analysis.

### Study design

The study consisted of prospective cross-sectional field surveys for malaria from 2005 and 2008. Surveys were conducted in villages that were selected by simple random procedure each year, with individuals of all ages assembling at pre-arranged meeting points for malaria screening.

### Data and sample collection

**Figure 1:**
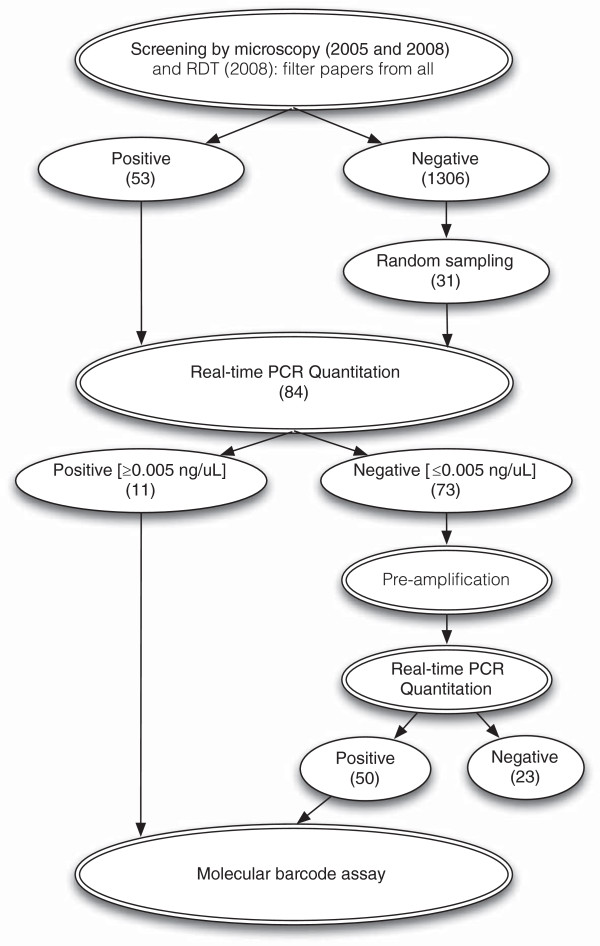
Flowchart of samples collected, processed and analyzed for this study using the molecular barcode assay.

Malaria screening was conducted by microscopy (2005 and 2008) or rapid diagnostic test (RDT, ICT Diagnostics South Africa, 2008 only, Figure 1). Microscopy included thick and thin blood film analysis performed later at the MIAM laboratory each screening day or the following morning. All individuals found positive by RDT (in 2008) or later by microscopy (in 2005 and 2008) were treated using artemether-lumefantrine following national guidelines, irrespective of whether or not they had symptoms. Dry blood spots (DBS) were collected on filter paper from all patients for subsequent parasite genotyping analysis.

### Sample pre-amplification

*Plasmodium falciparum* DNA was extracted from DBS samples using the Promega DNA IQ Casework Pro Kit for Maxwell 16 (Promega Corp., Madison, WI, USA) following the manufacturer’s instructions. Real-time DNA quantification was performed on resultant sample extracts to determine DNA content, as described by Daniels *et al.*[6]. Briefly, 3 μL of each extracted sample was used to determine the parasite template concentration in triplicate using a TaqMan assay designed to amplify PF3D7_0718800 (formerly PF07_0076), a single-copy conserved protein with unknown function. Lab-adapted *P. falciparum* strains were used as standards in the quantitative real-time reaction with a total reaction volume of 5 μL.

The *P. falciparum* infection samples from 2005 and 2008 were subjected to polymorphic analysis using a 24 SNP-based parasite molecular barcode assay on an ABI 7900 real-time PCR system, also as described by Daniels *et al.*[6]. The molecular barcode is a set of 24 SNP genotyping assays selected from across the *P. falciparum* genome to be selectively neutral; concatenating the genotypes from these independent assays generates what is known as the molecular barcode. Samples from low-grade infections, which gave DNA concentrations below the detectable threshold for the barcode assay (0.005 ng/μL), were subjected to pre-amplification (PreAmp Master Mix, Life Technologies, Inc, Grand Island, NY, USA) to enhance the template concentration specifically in the regions interrogated by the molecular barcode, following the manufacturer’s instructions. Pre-amplification reactions comprised 5 μL template DNA extract, 1X TaqMan assay mixture containing 0.2x TaqMan barcoding and quantification primers, and 1X Preamp master mix in 20 μL total volumes. Following pre-amplification, samples were re-quantified as described above.

Several other master mixes were also evaluated to determine which offered the best performance, testing a master mix from another vendor (Roche cDNA Pre-Amp Master, Roche Applied Science, Mannheim, Germany) and a standard genotyping master mix (Life Technologies TaqMan Universal PCR Master Mix, No AmpErase UNG) under the same pre-amplification thermal cycler conditions described above.

### Data analysis

Differences in DNA yields from field DBS extracts for protocols with and without pre-amplification were tested using McNemar’s Chi-square and the paired *t*-test for quantitative data. Temporal changes in parasitaemia and demographic profiles were examined using Student’s *t*-test and Mantel-Haenzel’s Chi-square, respectively.

## Results

### Background characteristics

There were no significant differences in human demographic characteristics for field samples from 2005 and 2008. However, *P. falciparum* infections exhibited substantially lower parasite densities in 2008 compared to 2005 (Table 1). All samples were from asymptomatic carriers in the resident population.

**Table 1:** **Parasite densities from *Plasmodium falciparum *** **samples and corresponding host characteristics**

	**2005**	**2008**	***p-value***
**GMPD (/μL)**	704	39	< 0.0005
**[95% CI]**	[390–1,271]	[23–68]	
**Parasite density (/μL) range**	35–48,569	8–171	
**Mean axillary temperature (°C)**	36.4	36.6	0.239
**[95% CI]**	36.2–36.6	[36.3–36.8]	
**Mean age (years)**	9	13	0.08
**[95% CI]**	[8.2–10.7]	[5.0–21.9]	
**Males (%)**	52	27	0.138

(GMPD, geometric mean parasite density [for positives only, n = 42 and 11, respectively for 2005 and 2008]; for all samples n = 53 and 31, respectively in 2005 and 2008).

### *Plasmodium falciparum* DNA yields from field samples with and without pre-amplification

With declining parasite densities between 2005 and 2008, an increasing proportion of samples could not be directly genotyped after extraction since yields of *P. falciparum* DNA concentrations fell below the 0.005 ng/uL threshold [6] for reliable barcode assay analysis (Table 2). This constraint was severe among samples from 2008 as compared to 2005, with both lower average DNA concentration, and more specimens yielding no direct DNA signal among 2008 samples (Table 2), consistent with declining parasite densities (Table 1).

**Table 2:** DNA yield from standard extraction among 2005 and 2008 DBS samples (n = 84)

	**2005**	**2008**	***p*-value**
**Samples with insufficient DNA signal (n)**	79.2% (42/53)	100% (31/31)	<0.0005
**Mean DNA concentration (ng/μL) in extracts [95% CI]**	0.0003068	0.000042	<0.0005

However, application of pre-amplification led to substantially more samples with sufficient DNA concentrations to yield a DNA signal (Table 3). Of the 84 field samples tested, 53 (63%) were microscopy-positive. Even among microscopy-positive samples, 30 (35.7% overall) failed standard qPCR. With pre-amplification, the number of untypable low parasitaemia samples was reduced to 23 (27.3% overall). Furthermore, of 31 samples negative by RDT or microscopy as well as by qPCR, nine yielded a molecular barcode after pre-amplification. Quality control measures (negative no-template controls, repeated trials with the original sample material and unique barcode results for the amplified samples) ruled out cross-contamination to explain this observation. Although all reasonable precautions were taken in the field and in the laboratory, we cannot completely rule out the possibility that any of these samples were mis-labeled; however, our detection threshold experiments indicate that our observation of molecular barcodes from samples considered to be negative by standard methodologies is likely to be valid.

**Table 3:** DNA yields from dried blood spot field samples before and after pre-amplification

	**Before pre-amplification**	**After pre-amplification**	***p*-value**
**Samples with insufficient DNA signal (%)**	73 (86.9%)	23 (27.3%)	<0.0005
**Mean DNA concentration (ng/μL) in extracts**	0.0001712	0.1301491	<0.0005
**[95% CI]**	[0.0001089-0.0002335]	[0.0864078-0.1738905]	

### Detection thresholds

The limits of detection for this pre-amplification approach were determined using serial dilution of standard DNA samples. Pre-amplification of samples resulted in reliable barcode data among samples containing as low as 0.00015 ng/μL DNA. Without pre-amplification, the lowest detectable sample DNA concentration for reliable barcode detection required 100X greater DNA concentration (0.015 ng/μL) (see Additional files 1 and 2). This threshold was higher than the 0.005 ng/μL determined in early studies using an older rotary glass capillary-based real-time PCR machine. Three of nine submicroscopic samples successfully typed after pre-amplification were also negative by standard nested PCR.

The method of pre-amplification was further evaluated by testing the performance of an additional pre-amplification mix from another vendor as well as the standard molecular barcode genotyping mix under the same pre-amplification conditions. The Roche cDNA Pre-Amp Master also amplified templates at concentrations lower than the standard limit of detection for the molecular barcode assay, but had a higher threshold to produce a complete molecular barcode compared to the Applied Biosystems product (0.001 ng vs. 0.00015 ng). The standard genotyping master mix did not improve the product yield compared to both of these pre-amplification products (see Additional file 3).

## Discussion

As malaria recedes under scaled-up interventions applied in endemic countries, the formidable scourge of malaria is increasingly assuming subpatent infections in semi-immune, asymptomatic segments of the population [7]. These submicroscopic infections pose a new challenge, not only as a reservoir for possible resurgence, but also for detection, documentation and tracking of intervention impact on the parasite.

The current study shows that the average parasite density of *P. falciparum* parasitaemia in the Macha area of southern Zambia decreased substantially between 2005 and 2008. There was a clear preponderance of submicroscopic, and hence sub-RDT, infections in 2008, which were much less amenable to detection and genotyping than samples from 2005.

Here the application of a TaqMan pre-amplification assay demonstrated that this approach significantly increased the proportion of low-parasitaemia field samples that could undergo molecular genotyping, leading to successful analysis of subpatent *P. falciparum* using a molecular barcode technique. More work will be required to substantiate the observations reported here, and to understand the impact of asymptomatic carriage, for example, on persistent malaria infection and transmission. Although other methodologies, such as nested PCR, can amplify DNA signals from low-yield samples, these approaches only allow amplification of one gene at a time. Pre-amplification, on the other hand, works with up to 100 short (less than 150 bp) amplicon assays in a single reaction, thus allowing genotyping of multiple loci. The amplification conditions, primarily the low primer concentration, limited number of amplification cycles, and increased extension time, together reduce chimera formation and maintain sample integrity. This strategy is not specific to TaqMan-based assays, and pre-amplification has been successfully incorporated for used in gene-specific assays as well as high-resolution melting assays for drug resistance genotyping [8].

By overcoming the detection challenge posed by these low-grade silent infections, pre-amplification may aid in monitoring intervention efficacy, tracking of the last bastions of residual parasites and thwarting potential resurgences during malaria control and elimination programs. As malaria-endemic regions reduce malaria transmission and work toward elimination, it will become critical to have tools and strategies to identify and track asymptomatic carriers, with highly reduced parasitaemia, that contribute to incident infections.

## Conclusions

The TaqMan pre-amplification assay provides a means for sensitive detection and tracking of residual *P. falciparum* infections during malaria control and elimination programmes.

## Abbreviations

DBS: Dried blood spot; DNA: Deoxyribo nucleic acid; LEV: Low elution volume; PCR: Polymerase chain reaction; SNP: Single nucleotide polymorphism; ABI: Applied Bioystems Inc; CI: Confidence interval; RBM: Roll Back Malaria; PMI: US President’s Malaria Initiative; MACEPA: Malaria Control and Evaluation Partnership; MIAM: Malaria Institute at Macha; RDT: Rapid diagnostic test; qPCR: quantitative PCR; bp: base pair; GMPD: Geometric mean parasite density.

## Competing interests

The authors declare that they have no competing interests.

## Authors’ contributions

SM participated in the study planning, design, field data collection, laboratory assays, and performed data analysis and writing of the manuscript. RD introduced the pre-amplification assay and developed the protocol as well as ran laboratory assays and contributed to the manuscript. AS was a co-investigator and contributed genomics technical guidance as well as manuscript write up. DW provided guidance for the project. PT was the local field site project director and contributed to the manuscript. SV planned the science, supervised the molecular assays and provided overall guidance of the research and manuscript write up. All authors read and approved the final manuscript.

## Supplementary Material

Additional file 1 **Real-time PCR cycle threshold (Ct) values for barcode SNPs, at decreasing concentrations of input *Plasmodium falciparum *3D7 DNA, either with or without pre-amplification.** Eight samples (numbered 1 through 8) of 3D7 DNA at variable input concentrations ranging from 15 to 0.0000015 nanograms (ng) are shown with or without pre-amplification (PA). Resulting Ct values for each molecular barcode assay (numbered 1 through 24) are shown with UD (undetermined) indicating that a Ct value could not be determined due to low amplicon signal. Click here for file

Additional file 2 **Genotyping results for barcode SNPs, at decreasing concentrations of input *Plasmodium falciparum* 3D7 DNA, either with or without pre-amplification.** Eight samples (numbered 1 through 8) of 3D7 DNA at variable input concentrations ranging from 15 to 0.0000015 nanograms (ng) are shown with or without pre-amplification (preamp). Resulting calls for each molecular barcode assay (identified by SNP assay chromosome and coordinate) are shown with a “dash” (-) indicating that the genotyping call could not be determined for that assay. “Blank” indicates a no template control. Click here for file

Additional file 3 **Genotyping results from alternative pre-amplification kits.** Amplification of the 24 molecular barcode assays (identified by chromosome and coordinate number) from control samples (amplified using TaqMan Universal PCR Master Mix, no AmpErase UNG) or Roche samples (amplified using the Roche Realtime Ready pre-amp Master Mix) at various concentrations of input 3D7 *P. falciparum* DNA ranging from 1 to 0.0001 nanograms (ng). A “dash” (-) indicates that the genotyping call could not be determined for that assay. Click here for file
